# Involvement of the Heat Shock Protein HtpG of *Salmonella* Typhimurium in Infection and Proliferation in Hosts

**DOI:** 10.3389/fcimb.2021.758898

**Published:** 2021-11-16

**Authors:** Tao Dong, Weiwei Wang, Minhao Xia, Shujie Liang, Guangzhong Hu, Hui Ye, Qingyun Cao, Zemin Dong, Changming Zhang, Dingyuan Feng, Jianjun Zuo

**Affiliations:** ^1^ College of Animal Science, South China Agricultural University, Guangzhou, China; ^2^ Guangdong Provincial Key Laboratory of Animal Nutritional Control, Guangzhou, China

**Keywords:** *Salmonella* Typhimurium, HtpG, RNA-seq, infection, immunity

## Abstract

*Salmonella* Typhimurium is a common pathogen infecting the gastrointestinal tract of humans and animals, causing host gastroenteritis and typhoid fever. Heat shock protein (HtpG) as a molecular chaperone is involved in the various cellular processes of bacteria, especially under environmental stress. However, the potential association of HtpG with *S.* Typhimurium infection remains unknown. In this study, we clarified that HtpG could also play a role as an effector in *S.* Typhimurium infection. RNA-seq indicated that the flagellar assembly pathway, infection pathway, and chemotaxis pathway genes of *S.* Typhimurium were downregulated after the mutation of HtpG, which resulted in compromises of *S.* Typhimurium motility, biofilm formation, adhesion, invasion, and inflammation-inducing ability. In addition, HtpG recombinant protein was capable of promoting the proliferation of *S.* Typhimurium in host cells and the resultant inflammation. Collectively, our results illustrated an important role of HtpG in *S.* Typhimurium infection.

## Introduction

*S.* Typhimurium is a Gram-negative bacterium from Enterobacteriaceae, which can colonize the intestine of humans and a variety of animals (e.g., pigs, chickens, and cattle), causing gastroenteritis (Majowicz et al., 2010). *Salmonella* enters the digestive tract through mouth, invades into the epithelial cells of mucosal layer of the small intestine and Peyer’s Patches (PP), or enters the intestinal epithelial cells through the absorption of small intestinal villi epithelial cells (Monack et al., 2000). *Salmonella* can also be directly swallowed by dendritic cells (DCs) in the lamina propria (LP) of the small intestine epithelium (Tam et al., 2008). After invading host cells, *Salmonella* can survive in DCs or macrophages in PP or LP, and then rapidly spread through the reticuloendothelial cell system and colonize the liver and spleen (Haraga et al., 2008), followed by spread throughout the body through the blood, causing various symptoms such as diarrhea, vomiting, fever, and abdominal pain (LaRock et al., 2015).

Heat shock protein 90 (HSP90/HtpG), a genetically conserved member of the heat shock protein family found in eukaryotes and prokaryotes, is involved in a variety of cellular processes including protein folding, repair, and signal transduction (Grudniak et al., 2018). HtpG belonging to the HSP90 family of bacteria has been shown to be essential for maintaining the *E. coli* CRISPR/Cas3 System (Yosef et al., 2011), which is an important defense mechanism for prokaryotes against viruses and horizontal transfer of DNA and RNA (Barrangou et al., 2007; Marraffini and Sontheimer, 2008). Because HtpG has ATPase activity (Jin et al., 2017), the HtpG mutation influences many physiological processes of *Pseudomonas aeruginosa*, including activity of LasA protease, biofilm formation, motility, and amount of rhamnolipid and pyoverdine/pyrubinin (Grudniak et al., 2018). Furthermore, recent studies indicated that HtpG is also implicated in the process of bacteria-induced pro-inflammatory responses (Huang et al., 2019). Silencing the HtpG gene of *Pseudomonas sinensis* could delay the onset time of epinephelus coioides, and reduce mortality and infection symptoms of host (Huang et al., 2019). HtpG also participates in the secretion of colibactin in *E. coli* (Garcie et al., 2016). Although HtpG was reported to be associated with *Salmonella* invasion and survival in porcine enterocytes and macrophages (Verbrugghe et al., 2015), few studies have reported the role of HtpG of *S.* Typhimurium in stimulating host immune response. In view of the harm of *S.* Typhimurium to humans and animals coupled with the role of HtpG in regulating *Salmonella* virulence, this study constructed strains of HtpG mutant *S.* Typhimurium to infect cells and mice, revealing the roles of the HtpG in the physiology of *S.* Typhimurium and its infection of host.

## Materials and Methods

### *S.* Typhimurium Strains and Their Growth Condition

*S.* Typhimurium strains (ATCC14028) purchased from the Guangdong Engineering and Technology Research and Development Center of Microbial Food Safety (Guangzhou, China) was used as a wild type (WT). HtpG mutant strains (ΔhtpG) were in-frame deletion mutant strains constructed using the λ-RED homologous recombination method (Datsenko and Wanner, 2000). The complement strains denoted as CΔhtpG were constructed by amplifying the HtpG gene fragment followed by connected to pBR-322 plasmid and electrotransformed into the ΔhtpG strains. All the strains, WT, ΔhtpG, and CΔhtpG were cultured in lysogeny broth (LB, Huankai, China) at 37°C, 200 r/min on a shaker.

### RNA-Seq and Analyses

The single colonies of the WT strains (*n* = 4) and ΔhtpG strains (*n* = 4) were respectively incubated in fresh LB medium at 37°C, 180 r/min in an air shaker overnight. The next day, the bacterial suspension was added to the fresh LB at a ratio of 1:100 and cultured on an air shaker until OD600 = 1. The supernatant was discarded by centrifugation, and the bacteria were washed twice with sterile PBS, followed by storage of the bacterial pellet at −80°C. The samples were sent to Novogene Bioinformatics Technology Co., Ltd (Beijing, China) to perform RNA sequencing and analysis.

The Agilent 2100 bioanalyzer was utilized to detect the total amount and integrity of RNA. The mRNA is randomly interrupted in the Fragmentation buffer, fragmented mRNA as a template, used random primers to synthesize the first strand of cDNA in M-MuLV reverse transcriptase, and synthesized the second strand under the action of DNA polymerase I. AMPure XP beads were applied to screen cDNA of about 70–420 bp, and PCR amplification was performed. AMPure XP beads were used again to purify the PCR products to obtain the library. After the library passed the quality inspection, Illumina sequencing was performed. The image data of the sequenced fragments measured by the high-throughput sequencer were converted into sequence data (reads) by CASAVA base recognition. Filter the original data to obtain clean data, including removing reads with adapters, removing reads with unidentifiable base information, and removing low-quality reads. The reference genome and annotation file were obtained in NCBI. Bowtie2 software was used to locate and analyze clean data. Gene expression analysis used HTSeq v0.6.1 to calculate the reading of each gene, calculate the FPKM of each gene based on the gene length, and map it to the reading of the gene by technology. DESeq2 R package (1.20.0) was used for differential expression analysis. The method of Benjamini and Hochberg was used to adjust the *p*-value to control the false discovery rate. Genes with adjusted *p*-values less than 0.05 found by DESeq2 were designated as expression differences. The GO enrichment analysis of differentially expressed genes was realized by GOseq R package software, and the KOBAS software analyzed the statistical enrichment of differentially expressed genes in the KEGG pathway.

### Motility Assays

The swimming motility of *S.* Typhimurium WT or ΔhtpG strains was determined on plates of LB medium solidified with Agar (Huankai, China) at a concentration of 0.5%. One microliter of fresh bacterial suspension was pipetted with OD600 = 1 into a plate, followed by incubation for 12 h at 37°C to record the diameter of growth circle.

### Quantitative Biofilm Formation Assay

Biofilm formation by *S.* Typhimurium WT or ΔhtpG strains in the wells of microtiter plates after 24 h of incubation was evaluated by crystal violet staining. Cultures were grown overnight at 37°C with shaking and then diluted 1:100 in fresh LB medium. Aliquots of 200 μl of these cultures were dispensed into the wells of 96-well microtiter plates and cultured at 30°C for 24 h. After discarding the medium, the adhering bacteria were washed three times with sterile PBS and stained with 0.5% crystal violet solution for 30 min, followed by washing with sterile PBS and drying. Thereafter, the stained biofilm was dissolved with glacial acetic acid and the absorbance was measured at OD570.

### Cell Lines

IPEC-J2 and RAW 264.7 cell lines were cultured in Dulbecco’s Modified Eagle’s medium (DMEM) (Thermo Fisher Scientific, USA) supplemented with 4 mM L-glutamine, 1 mM sodium pyruvate, and 10% fetal bovine serum (FBS, Gibco, USA) at 37°C 5% CO_2_.

### Adhesion, Invasion, Intracellular Proliferation Assay, and Cell Infection

IPEC-J2 was used for adhesion and invasion assay; IPEC-J2 and RAW 264.7 were used for intracellular proliferation assay. The cells were colonized with an equal number of the indicated WT, ΔhtpG, and CΔhtpG strains for 1 h (multiplicity of infection, MOI ≈ 100). For adhesion assay, the cells were washed three times with sterile PBS and then incubated for 10 min with PBS containing 0.5% Triton X-100 (v/v). For invasion assay, the cells were incubated for another 30 min in DMEM with gentamicin (100 μg/ml), washed, and incubated with PBS containing 0.5% Triton X-100 for 10 min; for intracellular proliferation and cell infection assay, the cells were incubated for an additional 30 min in DMEM with gentamicin (100 μg/ml), washed and cultured in DMEM containing 50 μg/ml gentamicin, followed by collection of cell lysates at 1 h, 2 h, 4 h, 8 h, 12 h, and 24 h. Serial 10-fold dilutions of cell lysates were plated on LB agar and incubated for 16 h to count the UFC of bacteria.

### Experimental Animals

Eighteen 6-week-old male BALB/c mice with similar body weight were randomly divided into three groups and used for the *S.* Typhimurium challenge test. The mice were fasted for 12 h before the challenge, and mice were subsequently gavaged with 100 μl of PBS containing 1×10^8^ CFU *S.* Typhimurium WT strains or ΔhtpG strains (treatment group) or the same amount of PBS (NC group), re-feeding after half an hour of gavage. Four days after infection with *S.* Typhimurium, the mice were sacrificed by cervical dislocation, and the serum was collected. Liver, spleen, and thymus were completely stripped and weighed. Mouse liver, spleen, and ileum tissues were collected and stored at −80°C for subsequent experiments. The animal study was reviewed and approved by the Animal Care and Use Committee of the South China Agricultural University (SCAU2019B142).

### RNA Preparation and qRT-PCR

Total RNA was extracted from S. Typhimurium, mice, and cells with Eastep Super RNA extraction kit (Promega, Shanghai, China). Approximately 2 μg of total RNA was reverse-transcribed using a M-MLV reverse transcriptase kit (Promega, Shanghai, China). The primers were synthesized by Tsingke (Guangzhou, China). Quantitative PCR was performed in 20μL reaction system with specific primers and AceQ qPCR SYBR Green Master Mix (Vazyme, Nanjing, China). The amplification was operated on the CFX96 Touch Real-Time PCR Detection System (Bio-Rad, California, USA). The relative expression of pig and mouse mRNA was normalized to GAPDH, and the mRNA of *S.* Typhimurium was normalized to gyrA. The expression of genes was analyzed by the method of 2^−ΔΔCt^. The sequence information of all the primers involved in this study is shown in **Table 1**.

**Table 1:** Primer sequences.

Species	Genes	Primer sequences (5’→3’)	Tm (°C)
Mouse	GAPDH	F: AGGTCGGTGTGAACGGATTTG	58
		R: TGTAGACCATGTAGTTGAGGTCA	
	IL-18	F: GACTCTTGCGTCAACTTCAAGG	58
		R: CAGGCTGTCTTTTGTCAACGA	
	IFN-γ	F: TGCTGATGGCCTGATTGTCTT	60
		R: ACAGCAAGGCGAAAAAGGATG	
	IL-1β	F: GAAATGCCACCTTTTGACAGTG	62
		R: TGGATGCTCTCATCAGGACAG	
	TNFα	F: CAGGCGGTGCCTATGTCTC	62
		R: CGATCACCCCGAAGTTCAGTAG	
Pig	IL-1β	F: GAGCTGAAGGCTCTCCACCTC	60
		R: ATCGCTGTCATCTCCTTGCAC	
	TNFα	F: TTCCAGCTGGCCCCTTGAGC	62
		R: GAGGGCATTGGCATACCCAC	
	IL-8	F: AGGACCAGAGCCAGGAAGA	58
		R: AGCAGGAAAACTGCCAAGAA	
	IL-18	F: TATGCCTGATTCTGACTGTT	58
		R: ATGAAGACTCAAACTGTATCT	
		R: GCAGCAGCCATGTACTCT	
	GAPDH	F: CAAGGCTGTGGGCAAGGTCATC	60
		R: TTCTCCAGGCGGCAGGTCAG	
*S.* Typhimurium	HtpG	F: CTGGGAGAAAATCAACAAGGC	58
		R: GGAAGAGTCGGTATGGGTGG	
	PrgJ	F: GGCAGGCGGTCAATATCAGGTC	60
		R: CCGTGGCAATCGCCGAACC	
	FliC	F: CTTGCTGGCGGTGCGACTTC	60
		R: ACACCTGCTGCTGTCAATGCG	
	SipB	F: GTATGGCAGGCGATGATTGA	58
		R: ATAAACACTCTTGGCGGTATCC	
	SopB	F: AGCGGGCGAGGCGGTAAG	58
		R: CCGGCTGGGTCAACGATTGC	
	SipA	F: GGCGTAACCAGCAAGAGCATTA	60
		R: ACCGTCGTGTCTGATTGTAAGG	
	GyrA	F: CGGGATACAGTAGAGGGATAGC	62
		R: TCACCAACGACACGGGCAGA	

### Quantification of Cytokines

The cell culture medium was collected after the strains infected the cells for 12 h. The ileal and spleen tissues were homogenized with PBS, the supernatant was then collected after centrifugation at 4000 r/min for 5 min. Cytokines were quantified with Enzyme-Linked Immunosorbent (ELISA) kits (Neobioscience, Shenzhen, China) according to the respective instructions. Briefly, the samples were added to the test well and incubated at 37°C for 90 min to bind the antibody. Then, the biotinylated antibody and avidin HRP were sequentially added to the test wells. Tetramethylbenzidine substrate solution is used for color development and stop solution to stop color development. Read the value at 450 nm with a microplate reader (Bio-Rad, California, USA).

### Recombinant Protein Purification

HtpG recombinant proteins containing endogenous His, S, and Trx tags derived from the pET-32a cloning vector (Novagen, Germany) were purified using a Ni-affinity column (Sangon, China). Briefly, double digestion of pET-32a with restriction enzymes *Nco*I and *Xho*I amplified the HtpG gene that repeats 20 nt with the restriction site of the vector; Trelief™ SoSoo Cloning Kit (Tsingke, Beijing) was applied to ligate the linearized plasmid with the amplified fragment. Then, the constructed plasmid was transformed into *E. coli* BL21 strain (Tsingke, Beijing). BL21 strain was grown in 1 L of LB broth within ampicillin (50 mg/ml) at 37°C for 3 h, and IPTG (1 mM) was used to induce at 20°C for 16 h. After enzymatic digestion consisting of 0.2 mg/ml lysozyme, 20 μg/ml DNase, 1 mM MgCl_2_, and 1 mM phenylmethylsulfonyl fluoride (PMSF), the supernatants were purified by Ni-column. The Toxin-Eraser Endotoxin Removal Kit (Genscript, USA) was used to remove lipopolysaccharide (LPS), and the Toxin-Sensor Chromogenic LAL Endotoxin Assay Kit (Genscript, USA) was used to detect the residual LPS. Ultrafiltration tube (Millipore, USA) was used for concentration and desalting. The protein concentrations were measured by Bradford assay (Thermo Fisher, USA). The purity was tested by SDS-PAGE (10%, acrylamide).

### Cell Proliferation and Toxicity Assay

Cell proliferation was determined using Cell Counting kit-8 (CCK-8, Dojindo, Japan). Add 100 μl of suspension containing about 1×10^4^ IPEC-J2 cells to a 96-well cell plate and culture it for 6 h. Then, add different concentrations of HtpG recombinant protein and culture it for 24 h. Add 10 μl of CCK-8, incubate for 1 h, and measure the absorbance at 450 nm. The LDH-assay kit (Nanjingjiancheng, China) was used to assess the cell toxicity.

### Statistical Analyses

All data are expressed as the means ± standard error (SE). Data analysis was performed using the SPSS 25.0 software (IBM), and one-way analysis of variance with Dunnett’s test was used. *p <* 0.05 was considered statistically significant. Comparison between the two sets of data was used with independent sample *t*-test, and differences were considered significant when *p < 0.05 and **p < 0.01 were obtained.

## Results

### Construction of HtpG Deletion and Complemented Strains of *S.* Typhimurium

**Figure 1:**
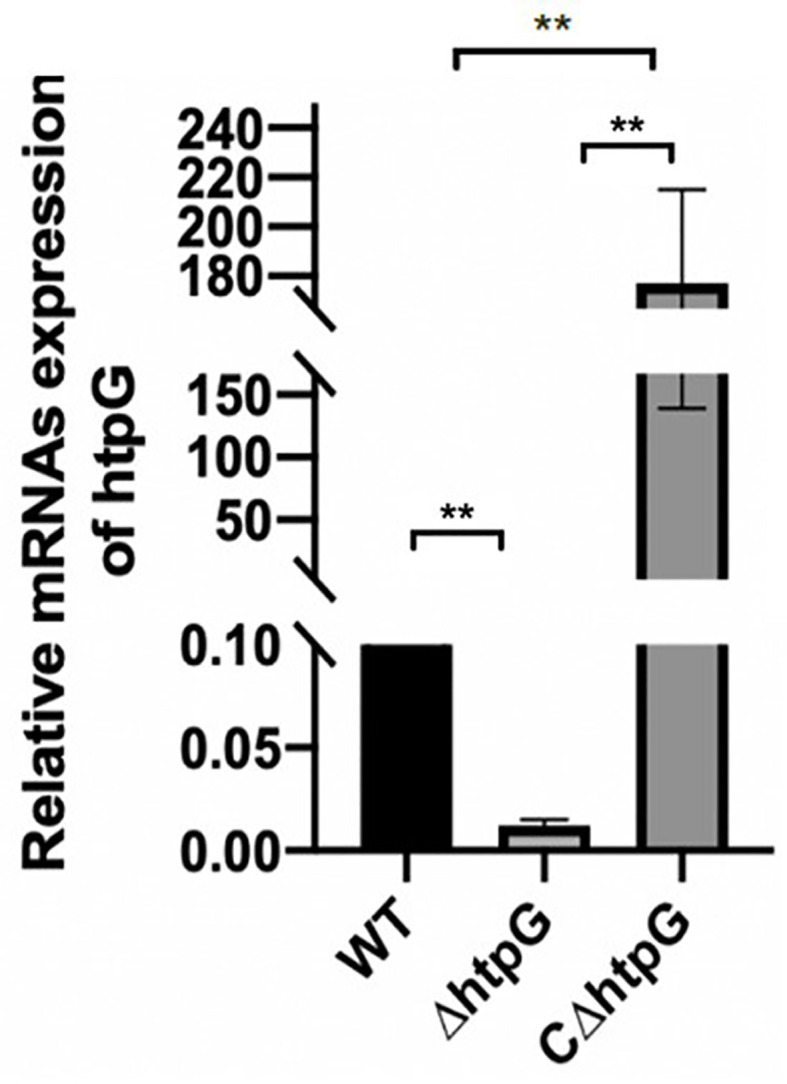
Relative expression level of HtpG mRNA of wild-type strains WT, mutant strains ΔhtpG, and complemented strains CΔhtpG; the result is shown as the mean ± standard error, ** means *p <* 0.01, *n* = 6.

To study the regulation of HtpG gene in *S.* Typhimurium, HtpG mutant strains (ΔhtpG) were constructed by RED homologous recombination system. Based on the mutant strains, the pBR-322 plasmid was used to construct complement strains (CΔhtpG). The results of qPCR showed that the relative expression of HtpG mRNA in mutant strains was significantly reduced (*p <* 0.01), while that of supplemented strains was significantly increased (*p <* 0.01) (**Figure 1**).

### RNA-Seq and Analysis of *S.* Typhimurium After HtpG Mutation

**Figure 2:**
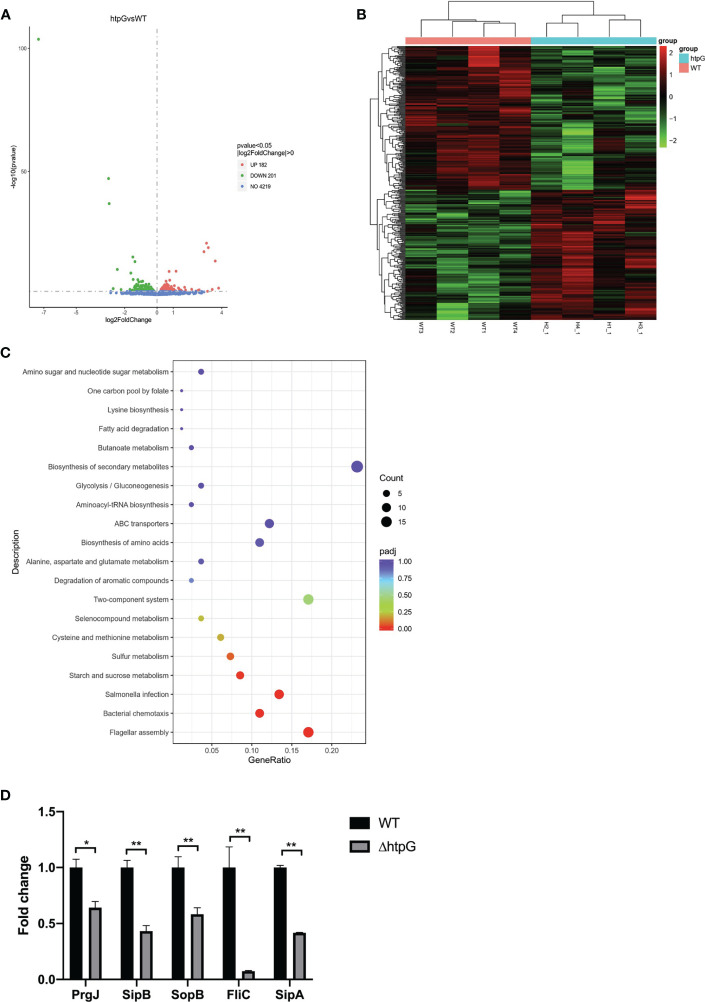
**(A)** Difference analysis volcano map. UP (red): upregulation of differential genes, DOWN (green): downregulation of differential genes, NO (blue): total number of genes detected. **(B)** Cluster Heat Map of Differential Genes. **(C)** KEGG enrichment analysis scatter plot. Gene ratio: the ratio of the number of differential genes annotated to the KEGG pathway to the total number of differential genes. **(D)** Transcriptomics verification by qRT-PCR, * means *p <* 0.05, ** means *p <* 0.01, *n* = 6.

To reveal the regulatory mechanism of HtpG, transcriptome sequencing and analysis of *S.* Typhimurium wild strains and HtpG-mutant strains were conducted. Overall, 6.45–8.18 million pieces of clean data and 1.0–1.2 Gb of RNA-seq data in wild-type strains and HtpG-mutant strains were obtained (**Table S1**). The data were submitted to the NCBI Sequence Reads Archive database (PRJNA746115). The reads obtained by sequencing were mapped to the *S.* Typhimurium genome (NC_003197.2) in the NCBI database. The results are shown in **Table S2**, the total mapped comparison rate of each sample is above 99%, and the multiple mapped comparison rate is above 95%. The analysis of gene expression differences was performed using DESeq2 software, and the screening criteria were *p <* 0.05. Compared with the WT strains, the ΔhtpG strains had a total of 383 differentially expressed genes, including 182 upregulated genes and 201 downregulated genes. (**Figures 2A, B**). According to KEGG pathway analysis in **Figure 2C**, the upregulated genes were mainly enriched in the ribosomal pathway, while the downregulated genes were mainly enriched in the *Salmonella* infection, flagellar assembly, bacterial chemotaxis, and other pathways.

Five differential genes enriched in *Salmonella* infection pathway were randomly selected for verification of the transcriptome result. Compared with the WT strains, the relative expression of PrgJ, SipB, SopB, FliC, and SipA mRNA of the ΔhtpG strains was significantly downregulated (*p <* 0.05), which was consistent with the results of transcriptomics (**Figure 2D**).

### Biological Characteristics of HtpG-Mutant Strains of *S.* Typhimurium

**Figure 3:**
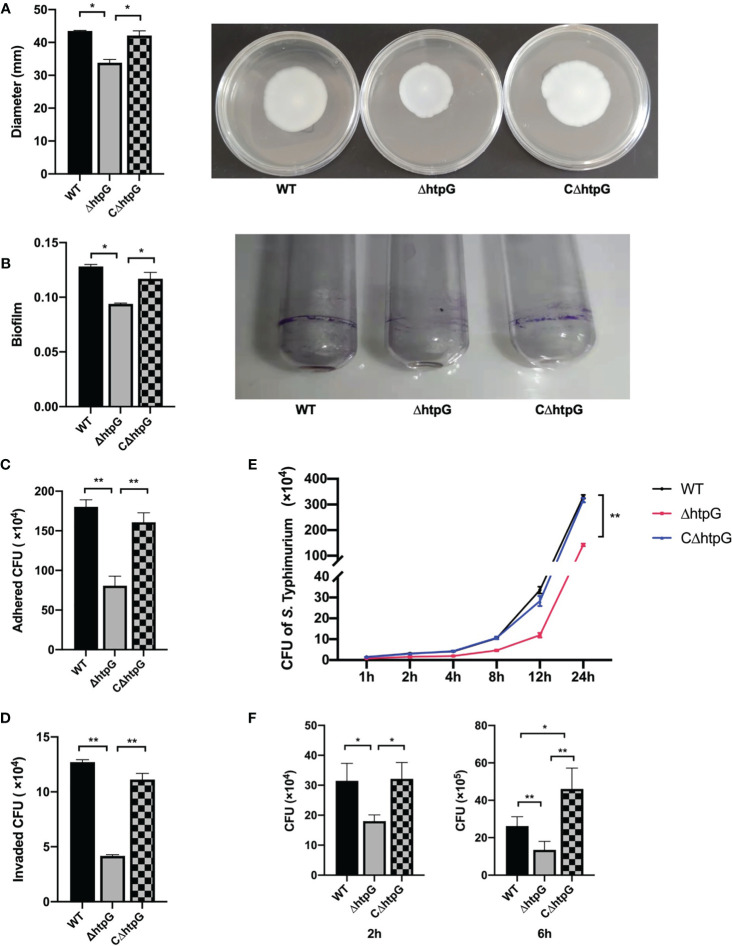
**(A)** Diameter of swimming circle of *S.* Typhimurium in semi-solid agar medium (left) and the representative graph (right), *n* = 4. **(B)** *S.* Typhimurium biofilm formation ability (left) and the representative graph: crystal violet stained the biofilm produced by *S.* Typhimurium in a glass tube (right), *n* = 6. **(C)** The number of *S.* Typhimurium attached to IPEC-J2 cells, *n* = 4. **(D)** The number of *S.* Typhimurium invading IPEC-J2 cells, *n* = 4. **(E)** The number of *S.* Typhimurium proliferating of WT strains, ΔhtpG strains, and CΔhtpG strains in IPEC-J2 cells, *n* = 4. **(F)** The number of *S.* Typhimurium proliferating in RAW264.7 cells, *n* = 4. The result is shown as the mean ± standard error, * means *p <* 0.05, ** means *p <* 0.01. WT means *S.* Typhimurium wild-type strains; ΔhtpG means *S.* Typhimurium HtpG mutant strains; CΔhtpG means *S.* Typhimurium HtpG complement strains.

RNA-seq results showed that flagellar assembly-related genes of *S.* Typhimurium were downregulated after HtpG mutation, which was then validated by determining the mobility of *Salmonella*, as represented by the size of the bacterial swimming circle. The results showed that the diameter of the swimming circle of ΔhtpG strains was significantly smaller than that of WT strains and CΔhtpG strains (*p <* 0.05) (**Figure 3A**).

The effect of HtpG mutation of *S.* Typhimurium on its biofilm formation ability was tested (**Figure 3B**). The results showed that the amount of biofilm formed by the ΔhtpG strains was significantly lower than that of the WT strains and CΔhtpG strains (*p <* 0.05).

Adhesion to intestinal epithelial cells is a key step of *S.* Typhimurium infecting the host (Wagner and Hensel, 2011). Thereby, IPEC-J2 cells were used as the object to detect the difference in adhesion ability of WT strains, ΔhtpG strains, and CΔhtpG strains. After 1 h of incubation, the number of adhering and invading *S.* Typhimurium was counted. The results showed that the adhesion and invasion ability of the ΔhtpG strains were both significantly lower than that of the WT strains and CΔhtpG strains (*p <* 0.05) (**Figures 3C, D**).

*S.* Typhimurium with MOI ≈ 100 was added to IPEC-J2 cells, followed by detection of the number of *S.* Typhimurium within the cells at 1 h, 2 h, 4 h, 8 h, 12 h, and 24 h of infection. Compared with the WT strains and the CΔhtpG strains, the number of *S.* Typhimurium in the cells of the ΔhtpG strains at 1 h, 2 h, 4 h, 8 h, 12 h, and 24 h of incubation was extremely significantly reduced (*p <* 0.01) (**Figure 3E**), which suggested that the deletion of HtpG can reduce the intracellular proliferation ability of *S.* Typhimurium.

Similar results were found in the RAW264.7 cell experiment. Two hours after infection, the number of *S.* Typhimurium in RAW264.7 cells in the ΔhtpG infection group was significantly lower than that in the WT infection group and the CΔhtpG infection group (*p <* 0.05). After 6 h, compared with the WT and CΔhtpG infection groups, the number of bacteria in the cells of the ΔhtpG infection group was significantly reduced (*p <* 0.01), while the number of bacteria in the CΔhtpG infection group was significantly higher than that in the WT infection group (*p <* 0.05) (**Figure 3F**), indicating that mutating the HtpG gene of *S.* Typhimurium can reduce its ability to proliferate in intestinal epithelial cells and macrophages.

### Mutant of HtpG Gene Reduced the Pro-Inflammatory Effects of *S.* Typhimurium *In Vitro*

Transcriptomics revealed that HtpG mutation led to downregulation of *Salmonella* infection pathway gene expression; we thus analyzed the host inflammatory response induced by HtpG mutant strains during the infection process through an *in vitro* infection model.

#### Pro-Inflammatory Responses of IPEC-J2 Cells

**Figure 4:**
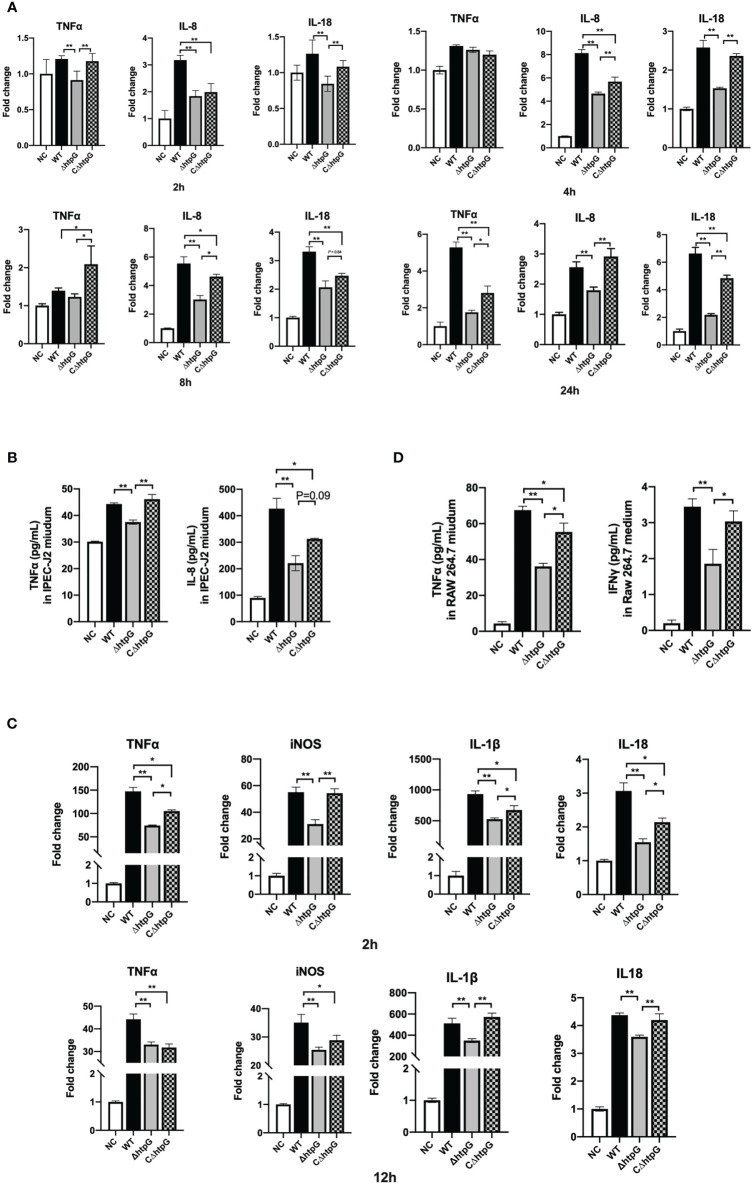
Pro-inflammatory responses of host cells induced by *S.* Typhimurium. **(A)** Relative mRNA expression of inflammatory cytokines in IPEC-J2 cells at different infection times of *S.* Typhimurium. **(B)** The contents of inflammatory cytokines in the cell culture medium of IPEC-J2 cells after 12 h of *S.* Typhimurium infection. **(C)** Relative mRNA expression of inflammatory cytokines in RAW264.7 cells at different infection times of *S.* Typhimurium. **(D)** The content of inflammatory cytokines in RAW264.7 cell culture medium after *S.* Typhimurium infection. These results are shown as the mean ± standard error, * means *p <* 0.05, ** means *p <* 0.01, *n* = 6.

As shown in **Figure 4A**, 2 h after infection with *S.* Typhimurium, the relative expression of TNFα and IL-18 in the cells infected with the ΔhtpG strains was significantly reduced compared with WT strain and CΔhtpG strain infection groups (*p <* 0.01). The relative expression of IL-8 in the ΔhtpG infection group and the CΔhtpG infection group was significantly lower than that in the WT infection group (*p <* 0.05). After 4 h of infection, there was no significant difference in the relative expression of TNFα between each group (*p >* 0.05). The relative expression of IL-18 and IL-8 mRNA in the ΔhtpG infection group was significantly lower than that in the WT infection and CΔhtpG infection groups (*p <* 0.01), and the relative expression of IL-8 in the CΔhtpG infection group was significantly lower than that in the WT infection group (*p <* 0.05). After 8 h of infection, the relative expression of TNFαin the CΔhtpG infection group was significantly higher than that of the other two groups (*p <* 0.05), while no significant difference was noted between the WT infection and ΔhtpG infection groups (*p >* 0.05). Compared with the WT infection group, the relative expression of IL-8 in the ΔhtpG infection group was significantly lower than that in the WT infection group (*p <* 0.01) and significantly lower than that in the CΔhtpG infection group (*p <* 0.05). After 24 h of infection, the relative expressions of TNFα, IL-8, and IL-18 mRNA in the ΔhtpG infection group were significantly lower than those in the WT infection and CΔhtpG infection groups (*p <* 0.05). The relative expression of IL-8 and IL-8 was significantly lower than that of the WT infection group (*p <* 0.05).

Besides the mRNA expression of inflammatory cytokines, the contents of inflammatory cytokines in the cell culture medium after 6 h of infection by *S.* Typhimurium in IPEC-J2 cells were determined. As shown in **Figure 4B**, the TNFα content of the ΔhtpG infection group was significantly lower than that of the WT infection and CΔhtpG infection groups (*p <* 0.01). The IL-8 content in the medium of the ΔhtpG infection group was significantly lower than that of the WT infection group (*p <* 0.01) and tended to reduce (*p* = 0.09) when compared with the CΔhtpG infection group, while the CΔhtpG infection group displayed a reduction (*p <* 0.05) in IL-8 content relative to the WT infection group (*p <* 0.05).

#### Pro-Inflammatory Responses of RAW264.7 Cells

Macrophages can actively swallow *Salmonella* that needs to survive and multiply in macrophages after breaking through the intestinal barrier to expand its infection of host. As shown in **Figure 4C**, after 2 h of incubation, the relative expression levels of TNFα, iNOS, IL-1β, and IL-18 in RAW264.7 macrophages infected with the ΔhtpG strains were significantly lower than those in the WT strains group (*p <* 0.01). Compared with the CΔhtpG group, the relative expression of TNFα, IL-1β, and IL-18 was significantly reduced (*p <* 0.05), the relative expression of iNOS was significantly reduced (*p <* 0.01), and the relative expression levels of TNFα, IL-1β, and IL-18 in the ΔhtpG group were significantly lower than those in the WT infection group (*p <* 0.05). After 12 h, the relative expression of TNFα, iNOS, IL-1β, and IL-18 in the ΔhtpG infection group was significantly lower than that in the WT infection group (*p <* 0.01), and the relative expression of IL-1β and IL-18 was extremely lower than that in the CΔhtpG infection group (*p <* 0.01). The relative expression of TNFα and iNOS in the CΔhtpG infection group was significantly lower than that in the WT infection group (*p <* 0.05).

With regard to cytokine contents in the cell culture medium (**Figure 4D**), the TNFα content of the ΔhtpG infection group was significantly lower than that of the WT and CΔhtpG infection groups (*p <* 0.01 and *p <* 0.05, respectively). The content of TNFα was significantly lower than that of the WT infection group (*p <* 0.05). The IFNγ content of the ΔhtpG infection group was significantly lower than that of the WT and CΔhtpG infection group (*p <* 0.01 and *p <* 0.05, respectively). No significant difference was observed between the CΔhtpG infection and WT infection groups (*p >* 0.05).

### Mutant of HtpG Gene Reduced the Pro-Inflammatory Effects of *S.* Typhimurium *In Vivo*

The final body weight of mice challenged with WT and ΔhtpG strains was both significantly lower than the mice in the NC group (*p <* 0.01); however, the final body weight of mice in the ΔhtpG strain group was significantly higher than the mice in the NC group (*p <* 0.05) (**Table 2**). This indicated that mutant of the HtpG gene can alleviate the weight loss of mice caused by *S.* Typhimurium. Compared with the NC group, the spleen and liver weights of the ΔhtpG group and the WT group were significantly increased (*p <* 0.05), and the weight of the thymus was significantly decreased (*p <* 0.05), indicating that the oral administration of *S.* Typhimurium caused huge damage to the mouse immune system. However, the ΔhtpG group exhibited a reduction (*p <* 0.05) of spleen weight along with an increase (*p <* 0.05) in final body weight relative to of the WT group (**Table 2**), which suggested that mutant of HtpG may reduce the toxicity of *S.* Typhimurium, probably attenuating host acute inflammation caused by its infection. To confirm this speculation, we then quantified inflammatory cytokines in mice after infection.

**Table 2:** The organ weight and body weight of the mice challenged with *S.* Typhimurium.

Groups	Thymus (g)	Liver (g)	Spleen (g)	Final body weight (g)
NC	0.053 ± 0.005^A^	1.067 ± 0.013^A^	0.08 ± 0.006^A^	24.1 ± 0.24^A^
WT	0.015 ± 0.002^b^	1.707 ± 0.010^b^	0.288 ± 0.004^b^	20.90 ± 0.26^b^
ΔhtpG	0.018 ± 0.002^b^	1.688 ± 0.024^b^	0.260 ± 0.006^c^	22.18 ± 0.09^c^

NC, negative control group, mice gavaged with PBS; WT, mice gavaged with WT strains; ΔhtpG, mice gavaged with ΔhtpG strains. Different letters in the same column indicate that the difference has reached a significant level of 0.05, and capital letters indicate that the difference has reached 0.01.

**Figure 5:**
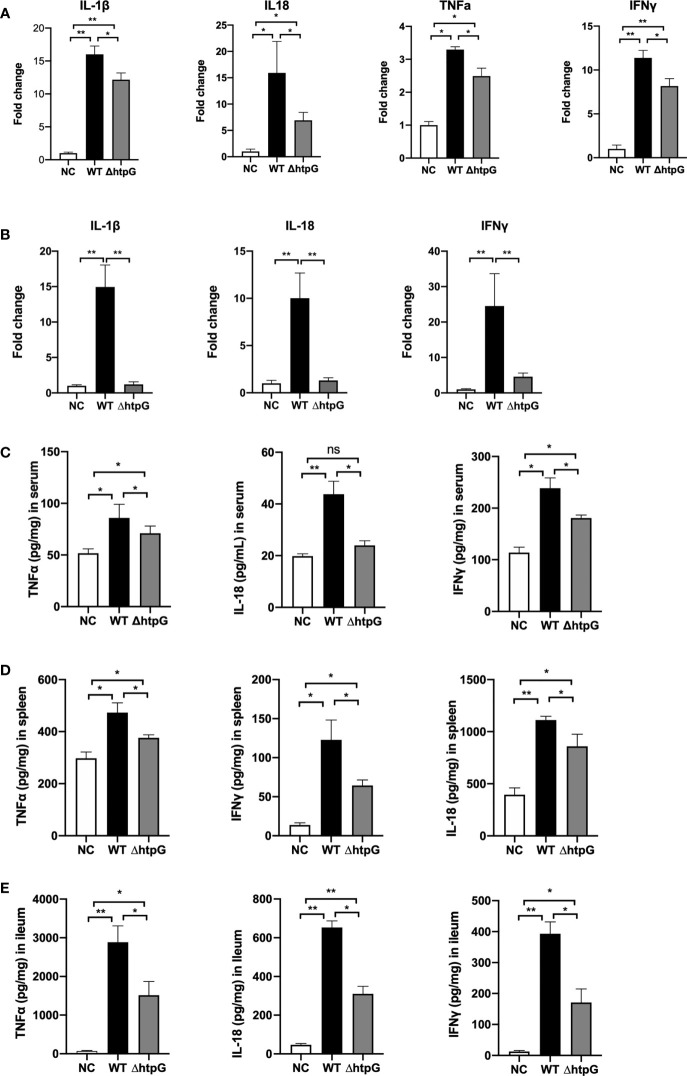
Pro-inflammatory responses of mice induced by *S.* Typhimurium. Relative mRNA expression in spleen **(A)** and ileum **(B)** after infection for 4 days with *S.* Typhimurium. The content of inflammatory cytokines in the serum **(C)**, spleen **(D)**, and ileum **(E)** of mice infected by *S.* Typhimurium. * means *p <* 0.05, ** means *p <* 0.01, *n* = 6.

As shown in **Figure 5A**, compared with the gavage with WT strains, the gavage with ΔhtpG strains decreased the relative expression of spleen IL-1β, IL-18, TNFα, and IFNγ of mice (*p <* 0.05). The relative expression of ileal IL-1β (*p <* 0.01) and IL-18 (*p <* 0.01) as well as IFNγ (*p <* 0.05) of mice gavaged with ΔhtpG strains was significantly lower than that gavaged with WT strains (**Figure 5B**). Regarding the inflammatory cytokine contents in mice after infection, the contents of TNFα, IFNγ, IL-1β, and IL-18 in serum, spleen, and ileum (**Figures 5C–E**) of mice challenged with ΔhtpG strains were significantly decreased (*p <* 0.05) as compared with mice challenged with WT strains. Overall, the mutation of HtpG gene could alleviate acute inflammatory injury in mice infected by *S.* Typhimurium.

### HtpG Protein Promotes *S.* Typhimurium Infection

**Figure 6:**
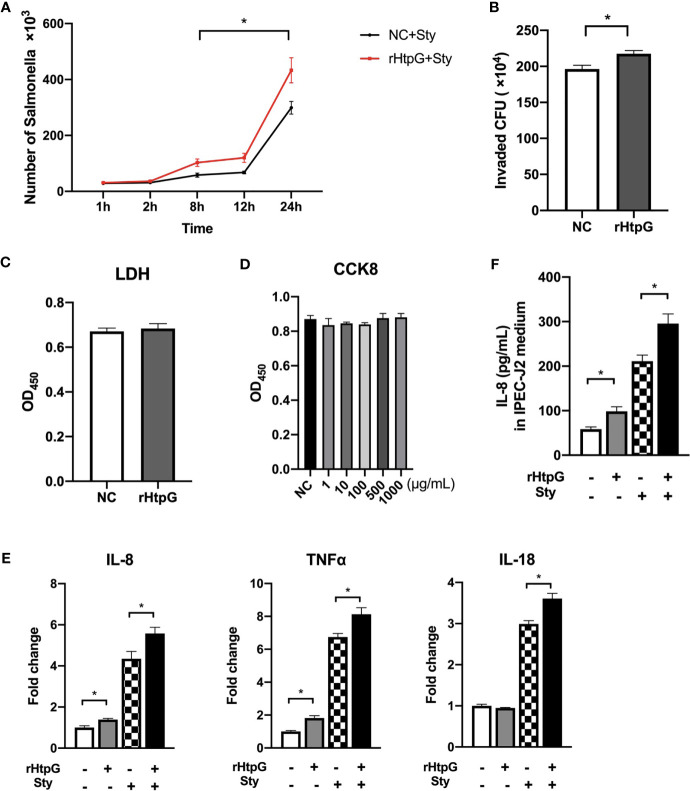
Influence of HtpG protein on *S.* Typhimurium infection. **(A)** Proliferation of *S.* Typhimurium in IPEC-J2 cells after rHtpG treatment, *n* = 4. **(B)** Number of *S.* Typhimurium infecting RAW 264.7 cells after rHtpG treatment for 6 h, *n* = 4. **(C)** The content of LDH in the cell culture medium after rHtpG treatment for 12 h, *n* = 6. **(D)** IPEC-J2 cell proliferation after rHtpG treatment, *n* = 6. **(E)** The relative mRNA expression of IPEC-J2 inflammatory cytokines, *n* = 6. **(F)** The content of IL-8 in IPEC-J2 cells medium after rHtpG treatment for 12 h, *n* = 6. rHtpG means HtpG Recombinant protein, Sty means *S.* Typhimurium, * means *p <* 0.05.

To further validate the role of HtpG in *S.* Typhimurium infection, a recombinant HtpG protein (rHtpG) derived from *S.* Typhimurium was constructed. It could be seen from **Figure 6A** that the addition of rHtpG had no significant effect on the proliferation of *S.* Typhimurium in IPEC-J2 cells at 1 h and 2 h of infection (*p >* 0.05). At 8 h, 12 h, and 24 h, the number of *S.* Typhimurium in the cells added with rHtpG was significantly higher than that in the control group (*p <* 0.05) (**Figure 6A**).

The number of *S.* Typhimurium in RAW 264.7 cells was detected 6 h after infection (**Figure 6B**). Compared with the control group, the number of *S.* Typhimurium in the rHtpG treatment group was significantly increased (*p <* 0.05).

The LDH content in the IPEC-J2 cell culture medium after adding rHtpG for 12 h was detected (**Figure 6C**). Compared with the control group, the LDH content in the rHtpG group did not increase significantly (*p >* 0.05), suggesting that the rHtpG would not cause cell damage. CCK-8 was used to detect cell proliferation for 12 h. As shown in **Figure 6D**, the absorbance of IPEC-J2 cells treated with different concentrations of rHtpG (1, 10, 100, 500, and 1000 μg/ml) did not change significantly compared with the control group, which revealed that rHtpG did not affect cell proliferation.

After incubating cells with *S.* Typhimurium and rHtpG for 12 h, we detected the relative mRNA expression and secretion level of inflammatory cytokines. As shown in **Figure 6E**, rHtpG significantly increased the relative expression of IL-8 and TNFα in IPEC-J2 cells (*p <* 0.05) but had no significant effect on IL-18 (*p >* 0.05) expression. In the case of *S.* Typhimurium infection, the addition of rHtpG could significantly increase the relative expression of TNFα, IL-8, and IL-18 (*p <* 0.05). Meanwhile, rHtpG significantly increased the secretion of IL-8 within cells (*p <* 0.05) (**Figure 6F**).

## Discussion

*S.* Typhimurium is a Gram-negative bacterium with a motility that can cause intestinal diseases in humans and manifold animals, including pigs, chickens, and cattle. In severe cases, it can cause sepsis and systemic infections (Desin et al., 2013). At present, the role of various effect factors of *S.* Typhimurium in the infected host has been fully explained (LaRock et al., 2015; Azimi et al., 2020; dos Santos et al., 2020); however, the role of chaperone proteins is frequently ignored. Heat shock protein 90 (HSP90/HtpG) belongs to the family of heat shock proteins, which is highly conserved genetically and widely found in eukaryotes and prokaryotes (Picard, 2002). It participates in a variety of cellular processes, including protein folding and repair and signal transduction, especially under external environmental stimuli such as heat stress (Dunner and Mason, 1999; Motojima-Miyazaki et al., 2010). It is generally believed that the lack of HtpG does not affect the growth of bacteria under conventional culture conditions (Thomas and Baneyx, 1998). However, under heat stress conditions, HtpG may have a wide range of regulatory effects on different prokaryotes (Schulz et al., 1997; Tanaka and Nakamoto, 1999; Hossain and Nakamoto, 2003).

In recent years, many studies have focused on the important role of HtpG in the process of bacterial infection. HtpG as a pathogenic factor contributes to the persistent infection of *Salmonella* in pigs (Verbrugghe et al., 2015). *Pseudomonas plecoglossicida* is a temperature-dependent pathogen, which is related to many diseases of fish. The HtpG mRNA was found to be significantly upregulated in a highly pathogenic status at 18°C. Besides, HtpG-RNAi strains exhibited lower toxicity (Huang et al., 2019), while there is currently no research to clarify how HtpG exerts its function in the process of *S.* Typhimurium infection. In this study, we constructed HtpG mutant strains and used RNA-seq to reveal the regulation of HtpG on *S.* Typhimurium effector factors, followed by exploration of the roles of HtpG in the *S.* Typhimurium infection of host through *in vivo* and *in vitro* infection models, thereby providing new ideas and theoretical basis for the prevention and treatment of *S.* Typhimurium.

Using λ-RED homologous recombination technology, we successfully constructed HtpG deletion strains. RNA-seq found that the downregulated genes of HtpG mutant strains were mainly implicated in the *Salmonella* infection pathway, flagella assembly pathway, and bacterial chemotaxis. A total of 14 downregulated genes, namely, FliC, FlgM, FliK, MotA, FlgN, FliD, FliA, FliN, FlgK, MotB, FliL, FlgH, FliJ, and FliY, were enriched in the flagella assembly pathway. As the only movement structure of flagella mediates the tropism of *Salmonella* (Barbosa et al., 2017), flagella also has antigenic properties triggering the host’s inflammatory response (Simon and Samuel, 2007). Thereby, the abnormal assembly of flagella induced by mutation of HtpG was deduced to lead to changes in biological characteristics related to the pathogenicity of *S.* Typhimurium. In support of this view, we found that the diameter of the swimming circle of the HtpG mutant strains was significantly lower than that of the WT strains.

Biofilm is an extracellular polymer formed by bacterial action on the surface of certain substances, including cellulose, bacterial protein components (fimbriae and flagella), lipids, and extracellular DNA (Merino et al., 2017), which is one of the reasons for the continuous infection of *Salmonella* (Desai et al., 2019). The great increases in the resistance of bacteria due to formation of biofilm is a crucial reason for the continuous contamination of *Salmonella* on biological products such as meat, eggs, and milk (Flemming and Wingender, 2010; Wang et al., 2020). We examined the biofilm formation of *S.* Typhimurium on cell culture plates made of polystyrene, which revealed that the biofilm formation ability of *S.* Typhimurium was significantly reduced by nearly 30% after the deletion of HtpG, indicating that HtpG is involved in the formation of *S.* Typhimurium biofilm. Grudniak et al. found similar results to us, for the HtpG mutant strain of *P. plecoglossicida*, and the migrating ability and biofilm formation ability were significantly downregulated (Grudniak et al., 2018).

We speculate that the reason for this phenomenon may be related to the downregulation of gene expression in the flagellar assembly pathway. Bacterial structures such as flagella and fimbriae play an important role in the process of approaching and adhering to the surface of the object, that is, the initial stage of biofilm formation (Milanov et al., 2017; Muhammad et al., 2020). Flagella-mediated movement and chemotaxis play a key role in the formation and maturation of *S.* Typhimurium biofilm (Steenackers et al., 2012). Pratt and Kolter (2010) proposed that in *E. coli*, flagella-mediated chemotaxis enables single cell to migrate to the surface of the attachment with sufficient nutrients; the motility mediated by flagella enables bacteria to reach the attachment surface at the beginning, overcome the electrostatic repulsion between the cells and the surface and attach to it; during the growth phase of the membrane, the movement ability helps the bacteria to spread along the surface, as well as promote the growth and extension of the membrane that continue to thicken (Pratt and Kolter, 2010). Flagella mutants (FlgE and FliC) of *S.* Typhimurium generate fewer biofilms in the early stage (Milanov et al., 2017). Huang et al. (2019) using RNA-seq revealed that the expression of FlgD and RplF virulence factors was downregulated, which was related to the reduction of biofilm yield, motility, and virulence of HtpG-RNAi strains (Huang et al., 2019).

We verified the pathogenic function of *S.* Typhimurium related to flagella adhesion and invasion of host cells. The number of HtpG-mutant strains that adhere to and invade into intestinal epithelial cells was significantly lower than that of wild strains, highlighting that HtpG could participate in the pathogenic function of *S.* Typhimurium in the adhesion and invasion of the host.

Verbrugghe et al. (2015) showed that 21 days post inoculation, *S.* Typhimurium (112910a phage type 120/ad, isolated from a pig stool sample) ΔhtpG strain was attenuated in ileocecal lymph nodes and cecal contents of pig *in vivo* (Verbrugghe et al., 2015). In IPEC-J2 cell invasion and intracellular proliferation experiments, they detected that the ΔhtpG strain has a decreasing trend, while we detected a significant decrease. The differences may be caused by different subspecies.

The downregulated genes enriched in the *Salmonella* infection pathway are FliC, SipB, SipC, SipA, SopB, PrgI, PrgJ, SpvC, and others. Among them, FliC is recognized as a pathogenic factor of *Salmonella* due to its role as a protein subunit of flagellar filaments. SipB, SipC, SipA, SopB, PrgI, PrgJ, and SpvC all belong to the SPI-1 virulence island coded and secreted effector proteins, depending on which *Salmonella* can effectively invade host cells; this explains why the HtpG mutant strains had reduced invasion and decreased intracellular proliferation.

In this study, the relative expression levels of inflammatory cytokines TNFα, IL-8, IL-18, and IL-1β in IPEC-J2 cells along with the secretion of TNFα and IL-8 in the cell supernatant were significantly lower in the ΔhtpG infection group than those in the WT infection group. A similar phenomenon was found for RAW264.7 macrophages responding to the mutation of HtpG. Furthermore, an *in vivo* experiment showed that the mRNA levels of inflammatory cytokines (e.g., IFNγ, IL-18, and IL-1β) in the ileum and spleen together with the levels of certain inflammatory cytokines (e.g., IFNγ, IL-18, and TNFα) in plasma, ileum, and spleen of ΔhtpG strain-challenged mice were significantly lower than those of WT strain-challenged mice, The similarity between the *in vitro* and *in vivo* results emphasized the compromised ability of *S.* Typhimurium following mutation of HtpG to induce host inflammation. The reason for this result may be related to the downregulation of the expression of related genes in the *S.* Typhimurium infection pathway after the HtpG mutation. Studies have shown that FliC (Ogushi et al., 2001; Franchi et al., 2006; Simon and Samuel, 2007), SipB (Hersh et al., 1999), SipC (Myeni and Zhou, 2010), SipA (Boyle et al., 2010), SopBv (Hu et al., 2017), PrgJ (Sukhan et al., 2003), and SpvC (Haneda et al., 2012) can induce host inflammation during *S.* Typhimurium infection. The stimulation of innate immune response by flagellin is critical to intestinal inflammation. *Salmonella* flagellin stimulates toll-like receptor (TLR)-5 of intestinal epithelial cells to induce IL-8 secretion through calcium-dependent NF-κB activation (Simon and Samuel, 2007). In macrophages, FliC and the rod-shaped protein PrgJ of the SPI-1 T3SS device activate caspase-1 (Sukhan et al., 2003). With the activation of caspase-1, the cells release pro-inflammatory cytokines IL-18 and IL-1β (Miao et al., 2010), subsequently promoting the release of IL-17 and IL-22 by T cells and expanding the inflammatory injury in the intestinal mucosa (Cho et al., 2012). SPI-1 effectors (including SopB, SopE, SopE2, SipA, SipC, and SopA) induce the production of pro-inflammatory cytokine IL-8 through MAPK and NF-κB pathways, triggering intestinal inflammation (Lou et al., 2019). Interestingly, SipB can directly activate caspase-1, mediating the activation of IL-18 and IL1-β of macrophages and inducing pyrolysis (Hersh et al., 1999).

To explore whether HtpG protein itself can be used as an effect factor to play a role in *S.* Typhimurium infection, we induced the expression of rHtpG by constructing a prokaryotic expression vector of HtpG. The purified rHtpG was then used to detect the role of HtpG in the infection of host by *S.* Typhimurium. As expected, rHtpG treatment of IPEC-J2 cells for 12 h could significantly increase the number of *S.* Typhimurium invading into cells and help *S.* Typhimurium proliferate within the cells. Besides, rHtpG treatment aggravated the inflammation of IPEC-J2 cells, as manifested by the increased expression and secretion of inflammatory cytokines. Shelburne et al. (2007) found that the rHtpG of *Porphyromonas gingivalis* can significantly upregulate the transcription and protein levels of CXCL8 (Shelburne et al., 2007). Mkl et al. indicated the rHtpG of *P. aeruginosa* can activate the NF-κB, CYLD, and MAPK pathways in a TLR4- and CD91-dependent manner, thereby stimulating the production of IL-8 in macrophages (Mkl et al., 2007). These studies show that the HtpG of a variety of bacteria can stimulate the secretion of IL-8 in the host, and we will continue to study the mechanism by which the htpG protein of *S.* Typhimurium induces host inflammation.

## Conclusion

Mutation of HtpG downregulates the expression of genes related to flagella assembly and infection of *S.* Typhimurium, leading to declines of its abilities of motility, biofilm formation, adhesion, and invasion, thus causing a decrease in host inflammatory response. The presence of HtpG protein can stimulate the inflammation of host intestinal epithelial cells and increase the infection effect of *S.* Typhimurium.

## Data Availability Statement

The datasets presented in this study can be found in online repositories. The names of the repository/repositories and accession number(s) can be found below: https://www.ncbi.nlm.nih.gov/, PRJNA746115.

## Ethics Statement

The animal study was reviewed and approved by the Animal Care and Use Committee of South China Agricultural University (SCAU2019B142).

## Author Contributions

JZ and DF designed and supervised the research work and guided the experiments. TD, WW, MX, SL, and GH conducted the animal and laboratory experiments and acquired the data. TD, HY, QC, CZ, and ZD analyzed the data and interpreted the results. TD and WW drafted the manuscript. JZ and DF revised the manuscript. All authors contributed to the article and approved the submitted version.

## Funding

This research is supported by the National Natural Science Foundation of China (4300-B18138).

## Conflict of Interest

The authors declare that the research was conducted in the absence of any commercial or financial relationships that could be construed as a potential conflict of interest.

## Publisher’s Note

All claims expressed in this article are solely those of the authors and do not necessarily represent those of their affiliated organizations, or those of the publisher, the editors and the reviewers. Any product that may be evaluated in this article, or claim that may be made by its manufacturer, is not guaranteed or endorsed by the publisher.
