# Supplementary material for: Involvement of the Heat Shock Protein HtpG of Salmonella Typhimurium in Infection and Proliferation in Hosts
Source: Front Cell Infect Microbiol. 2021 Nov 16;11:758898. doi: 10.3389/fcimb.2021.758898 (PMC8635147; doi:10.3389/fcimb.2021.758898)
Supplement: Supplementary file 1 [file Table_1.docx]

Table S1 Sequencing data quality

| Sample name | Raw reads | Clean reads | Clean bases | Error rate | Q20 | Q30 | GC content |
| --- | --- | --- | --- | --- | --- | --- | --- |
| H1 | 8252884 | 8181584 | 1.2G | 0.02 | 98.28 | 94.63 | 54.25 |
| H2 | 7699370 | 7639616 | 1.1G | 0.02 | 98.42 | 94.94 | 54.15 |
| H3 | 6512906 | 6453344 | 1.0G | 0.03 | 98.06 | 94.06 | 54.36 |
| H4 | 8217538 | 8158732 | 1.2G | 0.02 | 98.28 | 94.57 | 54.26 |
| WT1 | 7852476 | 7776776 | 1.2G | 0.02 | 98.28 | 94.62 | 54.42 |
| WT2 | 6818558 | 6750622 | 1.0G | 0.02 | 98.27 | 94.55 | 54.41 |
| WT3 | 7767642 | 7695878 | 1.2G | 0.02 | 98.35 | 94.73 | 54.35 |
| WT4 | 6881154 | 6807094 | 1.0G | 0.02 | 98.26 | 94.52 | 54.31 |

H means *S. typhimurium* HtpG mutant strains; WT means *S. typhimurium* wild type strains.

Table S2 Statistics of comparison between sample and reference genome

| Sample name | H1 | H2 | H3 | H4 | WT1 | WT2 | WT3 | WT4 |
| --- | --- | --- | --- | --- | --- | --- | --- | --- |
| Total reads | 8181584 | 7639616 | 6453344 | 8158732 | 7776776 | 6750622 | 7695878 | 6807094 |
| Total mapped | 8126197 (99.32%) | 7588643 (99.33%) | 6412987 (99.37%) | 8109479 (99.4%) | 7726768 (99.36%) | 6710995 (99.41%) | 7647124 (99.37%) | 6766357 (99.4%) |
| Multiple mapped | 266615 (3.26%) | 277256 (3.63%) | 224809 (3.48%) | 283034 (3.47%) | 272342 (3.5%) | 217202 (3.22%) | 262286 (3.41%) | 246396 (3.62%) |
| Uniquely mapped | 7859582 (96.06%) | 7311387 (95.7%) | 6188178 (95.89%) | 7826445 (95.93%) | 7454426 (95.85%) | 6493793 (96.2%) | 7384838 (95.96%) | 6519961 (95.78%) |
| Read-1 | 3931222 (48.05%) | 3656512 (47.86%) | 3095288 (47.96%) | 3914318 (47.98%) | 3728399 (47.94%) | 3247756 (48.11%) | 3693734 (48%) | 3260830 (47.9%) |
| Read-2 | 3928360 (48.01%) | 3654875 (47.84%) | 3092890 (47.93%) | 3912127 (47.95%) | 3726027 (47.91%) | 3246037 (48.09%) | 3691104 (47.96%) | 3259131 (47.88%) |
| Reads map to '+' | 3930000 (48.03%) | 3655569 (47.85%) | 3093978 (47.94%) | 3913216 (47.96%) | 3727214 (47.93%) | 3246846 (48.1%) | 3692181 (47.98%) | 3259868 (47.89%) |
| Reads map to '-' | 3929582 (48.03%) | 3655818 (47.85%) | 3094200 (47.95%) | 3913229 (47.96%) | 3727212 (47.93%) | 3246947 (48.1%) | 3692657 (47.98%) | 3260093 (47.89%) |
| Reads mapped in proper pairs | 7790510 (95.22%) | 7253394 (94.94%) | 6135276 (95.07%) | 7767588 (95.21%) | 7390140 (95.03%) | 6437084 (95.36%) | 7318484 (95.1%) | 6457874 (94.87%) |
| Proper-paired reads map to different chrom | 0 (0%) | 0 (0%) | 0 (0%) | 0 (0%) | 0 (0%) | 0 (0%) | 0 (0%) | 0 (0%) |
